# Effects of mating on female reproductive physiology in the insect model, *Rhodnius prolixus*, a vector of the causative parasite of Chagas disease

**DOI:** 10.1371/journal.pntd.0011640

**Published:** 2023-09-20

**Authors:** Jimena Leyria, Alessandra A. Guarneri, Marcelo G. Lorenzo, Marcela Nouzova, Fernando G. Noriega, Samiha A. M. Benrabaa, Francisco Fernandez-Lima, Lilian Valadares Tose, Ian Orchard, Angela B. Lange

**Affiliations:** 1 Department of Biology, University of Toronto Mississauga, Mississauga, Canada; 2 Instituto René Rachou, Avenida Augusto de Lima, Belo Horizonte, MG, Brazil; 3 Instituto de Investigaciones en Biodiversidad y Biotecnología (INBIOTEC-CONICET), Mar del Plata, Buenos Aires, Argentina; 4 Biology Center of the Academy of Sciences of the Czech Republic, Institute of Parasitology, České Budějovice, Czech Republic; 5 Department of Biological Sciences and Biomolecular Science Institute, Florida International University, Miami, Florida, United States of America; 6 Department of Parasitology, University of South Bohemia, České Budějovice, Czech Republic; 7 Department of Chemistry and Biochemistry and Biomolecular Science Institute, Florida International University, Miami, Florida, United States of America; Kenya Agricultural and Livestock Research Organization, KENYA

## Abstract

The blood-sucking hemipteran *Rhodnius prolixus* is one of the main vectors of Chagas disease, a neglected tropical disease that affects several million people worldwide. Consuming a blood meal and mating are events with a high epidemiological impact since after each meal, mated females can lay fertile eggs that result in hundreds of offspring. Thus, a better knowledge of the control of *R*. *prolixus* reproductive capacity may provide targets for developing novel strategies to control vector populations, thereby reducing vector-host contacts and disease transmission. Here, we have used a combination of gene transcript expression analysis, biochemical assays, hormone measurements and studies of locomotory activity to investigate how mating influences egg development and egg laying rates in *R*. *prolixus* females. The results demonstrate that a blood meal increases egg production capacity and leads to earlier egg laying in mated females compared to virgins. Virgin females, however, have increased survival rate over mated females. Circulating juvenile hormone (JH) and ecdysteroid titers are increased in mated females, a process mainly driven through an upregulation of the transcripts for their biosynthetic enzymes in the *corpus allatum* and ovaries, respectively. Mated females display weaker locomotory activity compared to virgin females, mainly during the photophase. In essence, this study shows how reproductive output and behaviour are profoundly influenced by mating, highlighting molecular, biochemical, endocrine and behavioral features differentially expressed in mated and virgin *R*. *prolixus* females.

## Author summary

Chagas disease is a neglected tropical disease transmitted to mammalian hosts primarily by blood-feeding insects named triatomines. The blood meal is required for triatomine females to initiate egg production. Virgin and mated females are capable of making and laying eggs but only the latter have a high epidemiological impact since after each meal, they lay fertile eggs that result in hundreds of offspring. *Rhodnius prolixus* is one of the most important triatomine vectors and historically, has been the model for studying insect physiology. Interestingly, although the size of the blood meal is the same for both virgin and mated *R*. *prolixus* females, mated females have increased egg production capacity. Here, we describe for the first time, molecular, biochemical, endocrine, and behavioral features that are differentially expressed in virgin and mated females of *R*. *prolixus* and that influence their reproductive physiology and behavior. A better understanding of triatomine reproductive capacity may provide targets for developing novel strategies to control vector populations, thereby reducing vector-host contacts and disease transmission.

## Introduction

Chagas disease, a zoonotic infection caused by the parasite *Trypanosoma cruzi*, is a neglected tropical disease that affects millions across Latin America; however, as a result of human migration, the disease is no longer confined to the tropics. It is currently recognised as a public health problem throughout the world, turning this infection into an emerging disease of growing epidemiological interest [1–3]. The parasite is mainly transmitted by blood feeding insects belonging to the Triatominae [4]. Active dispersal associated with searches for a source of blood or a mate are recognized among the most important mechanisms for house infestation [5–9]. *Rhodnius prolixus* Stål is not only one of the main Chagas vector species, but also a model for the study of insect physiology since the 1930s (see [10]).

Like all triatomines, this vector undergoes a hemimetabolous metamorphosis consisting of an embryo, five nymphal stages, and an adult phase. A blood meal is required to initiate molting in nymphs and full egg production in adults [11,12]. In triatomines, as oviparous organisms, embryonic development occurs in the egg and depends on the utilization of previously stored maternal material, namely yolk, synthesized mainly by the fat body (the main organ of intermediate metabolism in insects), which is then released into the hemolymph and taken up by the oocytes [13]. The process of yolk production and deposition is termed vitellogenesis, which represents a phase of accelerated egg growth leading to the production of mature eggs (oogenesis). Vitellogenesis is coupled to finely synchronised endocrinological events that promote successful oogenesis, and ovulation and oviposition [14,15].

In insects, there are two main types of lipophilic hormones; ecdysteroids and juvenile hormones (JHs), which are well known for their critical roles in growth, development, and reproduction [16]. However, although there is a paradigm on their molecular actions during reproduction (mainly related to hormone receptors and signaling pathways), the specific timing of action and role of these lipophilic hormones can vary between insect species [14]. JHs, a family of acyclic sesquiterpenoids produced and secreted from the *corpus allatum* (CA) [17], were originally postulated by Sir Vincent Wigglesworth to regulate successful reproduction in *R*. *prolixus* females [18]. Recently, we reported the participation of the homolog JH III skipped bisepoxide (JHSB_3_) and its signaling pathway via the Methoprene tolerant-Taiman-Krüppel homolog 1 (Met-Tai-Kr-h1) axis, in reproductive performance of *R*. *prolixus* females [19]. After a blood meal, mated females of *R*. *prolixus* increase their circulating JHSB_3_ titers [20], a process possibly modulated through allatoregulatory neuropeptides and insulin signaling pathway [19,21,22]. In female *R*. *prolixus*, ecdysteroids, a group of steroid hormones, control oocyte maturation and also ovulation, along with a mating factor, by promoting the release of a myotropic neurohormone from medial neurosecretory cells (MNSCs) in the brain [11,15,23].

A mated *R*. *prolixus* female can produce up to 30–40 eggs after a single blood meal [11,22,24], whereas virgin females make and lay fewer eggs [11,15]. From an epidemiological point of view, it is therefore essential to expand our understanding of *R*. *prolixus* reproductive capacity to provide targets for developing novel strategies to control the vector population and reduce vector-host contacts and disease transmission. This includes, for example, the identification of specific molecules for use in symbiont-mediated RNAi, a powerful technology which provides the potential for biocontrol against disease vectors, and which has already been tested in laboratory trials using *R*. *prolixus* [25]. Here, we have used a combination of gene expression analysis, biochemical assays, hormone measurements, and studies on locomotory activity to investigate how mating influences egg development and egg laying rates in females of *R*. *prolixus*. In essence, this study reaffirms the profound influence that mating has on female reproductive output, highlighting molecular, biochemical, endocrine and behavioral features that coordinate a full reproductive cycle in females of *R*. *prolixus*.

## Results

### At the end of the first reproductive cycle, egg production (eggs made and laid) is highest in mated females relative to virgins

**Fig 1:**
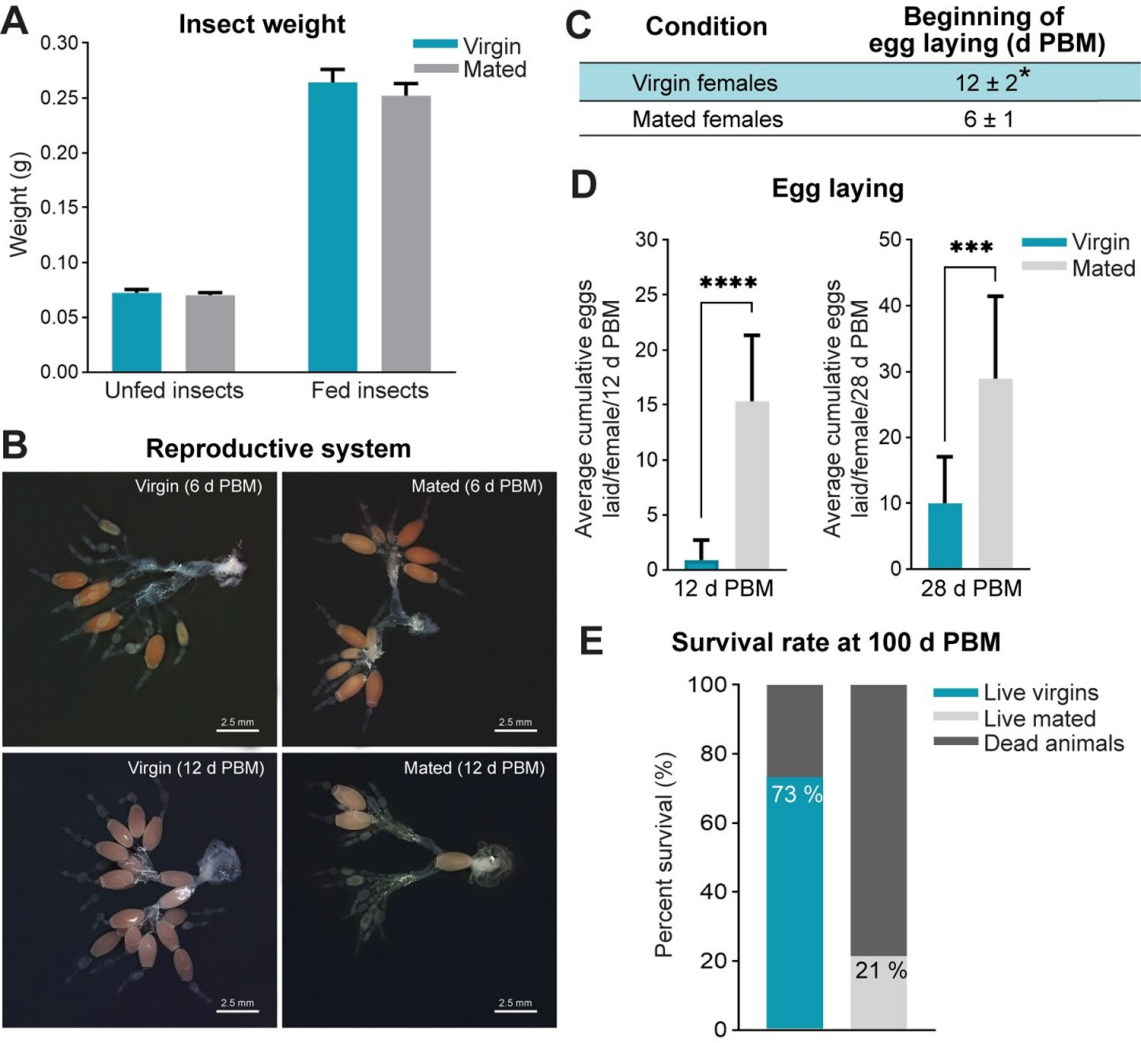
Reproductive physiology in fed virgin and mated females. **(A)** Weight of insects (grams) measured before (unfed insects) and immediately post blood meal (fed insects) (n = 15–20 females). **(B)** Representative images showing the reproductive system of virgin (left panel) and mated females (right panel) at 6 (upper) and 12 (bottom) days post blood meal (d PBM) (representative image of n = 15–20 females). **(C-D)** Parameters indicating reproductive performance in fed virgin and mated females. The beginning of egg laying in virgin (highlight in blue) and mated (highlight in white) females is shown in (C), and the average cumulative eggs laid per female at 12 (left) and 28 d PBM (right) is shown in (D). *p < 0.05; ***p < 0.001; ****p < 0.0001 (Student’s t-test, n = 15 females). **(E)** Survival rate at 100 d PBM shown as a percentage (n = 15 females).

It has previously been shown that egg production is highest in fed mated female *R*. *prolixus* compared to fed virgin females [26,27]. To confirm the effects of mating on egg production in our colony we fed both virgin and mated females (ten day post ecdysis) and monitored egg production. Since the size of a blood meal directly influences egg production, virgin and mated insects were weighed, fed, and re-weighed. Our results show that the blood meal size is not influenced by mating (Fig 1A). Since feeding is essential for full egg production, we then evaluated the ability of females to make and lay eggs. The ovarian morphology 6 days post blood meal (when egg laying typically begins) is comparable between virgin and mated females (Fig 1B, upper panel); however, more eggs remain retained in the ovaries, calyx and oviducts of virgin females relative to mated females 12 days post blood meal (virgin: 15 ± 3 eggs; mated: 3 ± 1 eggs) (Fig 1B, bottom panel). Indeed, the start of egg laying is delayed in virgins (12 ± 2 days post blood meal) relative to mated females (6 ± 1 days post blood meal) (Fig 1C). These effects are even clearer when the number of eggs laid is evaluated; the average of cumulative eggs laid per female throughout 12 days post blood meal is significantly lower in virgins when compared to mated females (virgin: 0.9 ± 1.8 eggs; mated: 15.33 ± 6 eggs; *p <* 0.0001) (Fig 1D, left panel). At the end of the first reproductive cycle (28 days post blood meal), each virgin female laid a total of ~ 10 ± 7.04 eggs, while each mated female laid ~ 28.9 ± 12.49 eggs (*p <* 0.001) (Fig 1D, right panel). When lifespan is evaluated 100 days post blood meal, virgin females survived significantly longer than mated females (73% vs. 21% alive, respectively, Fig 1E), supporting the most prevalent hypothesis concerning the trade-off between reproduction cost and longevity [28].

### Digestion in mated females is faster, resulting in increased protein and lipid production by the fat body

**Fig 2:**
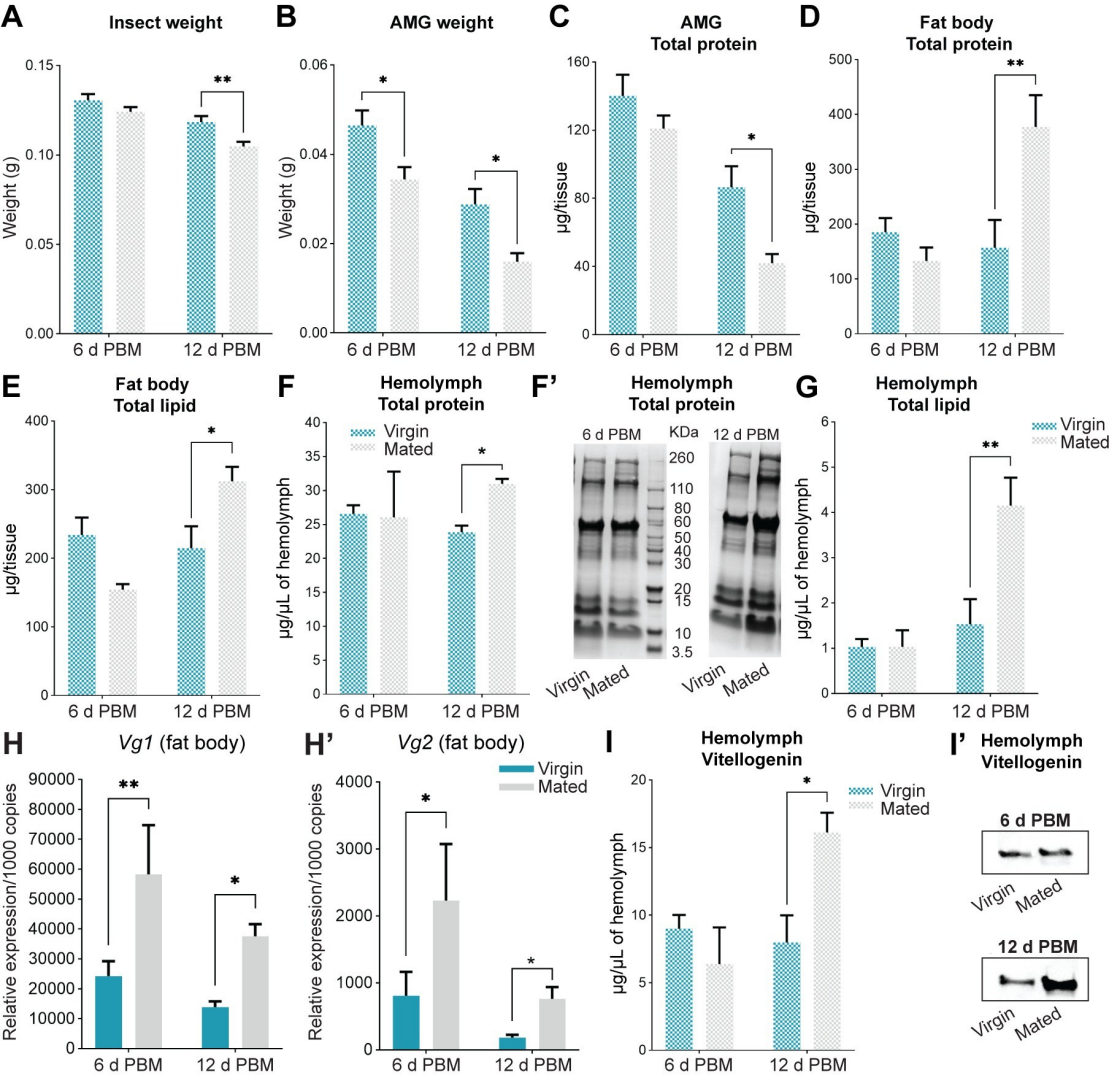
Blood meal size, anterior midgut weight, lipid, protein, and vitellogenin quantification in fed virgin and mated females. **(A)** Weight of insects (grams) was measured at 6 and 12 days post blood meal (d PBM); **p < 0.01 (Student’s t-test, n = 6–7 females). **(B-C)** Weight (B) and total protein content (C) in the anterior midgut (AMG) of virgin and mated females at 6 and 12 d PBM; *p < 0.05 (Student’s t-test, n = 6–7 independent biological replicates, where each n represents the AMG from 1 insect). **(D-E)** Total protein (D) and lipid content (E) in the fat body of virgin and mated females at 6 and 12 d PBM; *p < 0.05; **p < 0.01 (Student’s t-test, n = 6–7 females, where each n represents the fat body from 1 insect). **(F-F’)** Total protein (F) and SDS-PAGE analysis (F’) of the hemolymph (1 μL) of virgin and mated females at 6 and 12 d PBM; *p < 0.05 (Student’s t-test, n = 6–7 independent biological replicates, where each n represents hemolymph from 1 insect); in (F’) a representative image of 3 independent experiments is shown. **(G)** Total lipid circulating in the hemolymph of virgin and mated females at 6 and 12 d PBM; **p < 0.01 (Student’s t-test, n = 5 independent biological replicates, where each n represents hemolymph from 1 insect). **(H-H’)** *Vg1* (H) and *Vg2* (H’) mRNA expression in the fat body of virgin and mated females at 6 and 12 d PBM. Transcript levels were quantified using RT-qPCR and analyzed by the 2^-ΔCt^ method. The y axes represent the relative expression obtained via geometric averaging using *Rp49* and *actin* as reference genes. *p < 0.05; **p < 0.01; (Student’s t-test, n = 5–6 independent biological replicates, where each n represents an individual tissue from 1 insect). **(I)** Quantification of vitellogenin circulating in the hemolymph of virgin and mated females at 6 and 12 d PBM by ELISA. *p < 0.05 (Student’s t-test, n = 5–6 independent biological replicates, where each n represents hemolymph from 1 insect) **(I’)** Western blot image (1 μL of hemolymph in 1:20 dilution) showing Vg circulating in the hemolymph. A representative image of 3 independent experiments is shown.

In adult *R*. *prolixus*, mass increases 2 to 3 fold after a blood meal, and then decreases during digestion and excretion. Six days post blood meal, the weight of virgin and mated females is comparable, with a slight decrease in mated insects (Fig 2A); however, 12 days post blood meal, weight loss by mated females is significantly higher than virgins (*p* < 0.01; Fig 2A), in part due to virgin females retaining eggs. During the first day after feeding, weight loss is mainly caused by diuresis [29], and later by digestion of the blood meal, which begins some days after ingestion. In *R*. *prolixus*, blood proteins are kept undigested in the anterior midgut (AMG), and slowly transferred to the posterior midgut where digestion takes place [30]. The protein content of the AMG can be used as a proxy for the pace of blood meal digestion. Thus, we evaluated the emptying of the AMG by tissue weighing. At both time points evaluated, 6 and 12 days post blood meal, weight loss of AMG is higher in mated females (*p* < 0.05; Fig 2B), which also coincides with a lower protein content in the AMG, mostly seen 12 days post blood meal (*p* < 0.05; Fig 2C). As expected, we detect a significant protein (*p* < 0.01) and lipid (*p* < 0.05) increase in the fat body of mated females 12 days post blood meal, i.e., in the middle of vitellogenesis (Fig 2D and 2E, respectively). In agreement, at the same time point, total protein circulating in the hemolymph is higher in mated females when compared to virgins (virgin vs. mated: 23.88 ± 1.46 μg/μL vs. 30.96 ± 0.94 μg/μL; Fig 2F and 2F’), as well as hemolymph lipids (virgin vs. mated: 1.53 ± 0.73 μg/μL vs. 4.16 ± 0.73 μg/μL; Fig 2G). To evaluate whether lower egg production at the end of the first reproductive cycle in virgin females (Fig 1D, right) is related to a lower synthesis of the main yolk protein precursor (YPP), we measured transcript expression of vitellogenin (*Vg1* and *Vg2*) in the fat body. Although total protein in the fat body is only significantly increased 12 days post blood meal (Fig 2D), the results show that *Vg* mRNA levels are positively regulated after mating at both time points analyzed (Fig 2H and 2H’). However, in consonance with hemolymph total protein titers (Fig 2D), hemolymph Vg levels are significantly higher in mated females only at 12 days post blood meal (virgin vs. mated: 7.98 ± 2.64 μg/μL vs. 16.12 ± 1.87 μg/μL; Fig 2I and 2I’). These results point to the complex regulation linking digestion, protein and lipid synthesis (at the molecular and protein levels), and changes in the composition of the hemolymph. In fact, circulating protein titer not only depends on synthesis in the fat body, but also is the result of dynamic processes of protein uptake by the oocytes and protein degradation [31,32].

### Mating increases transcript expression of JH biosynthetic enzymes in the *corpus allatum*, as well as hemolymph JH titers

**Fig 3:**
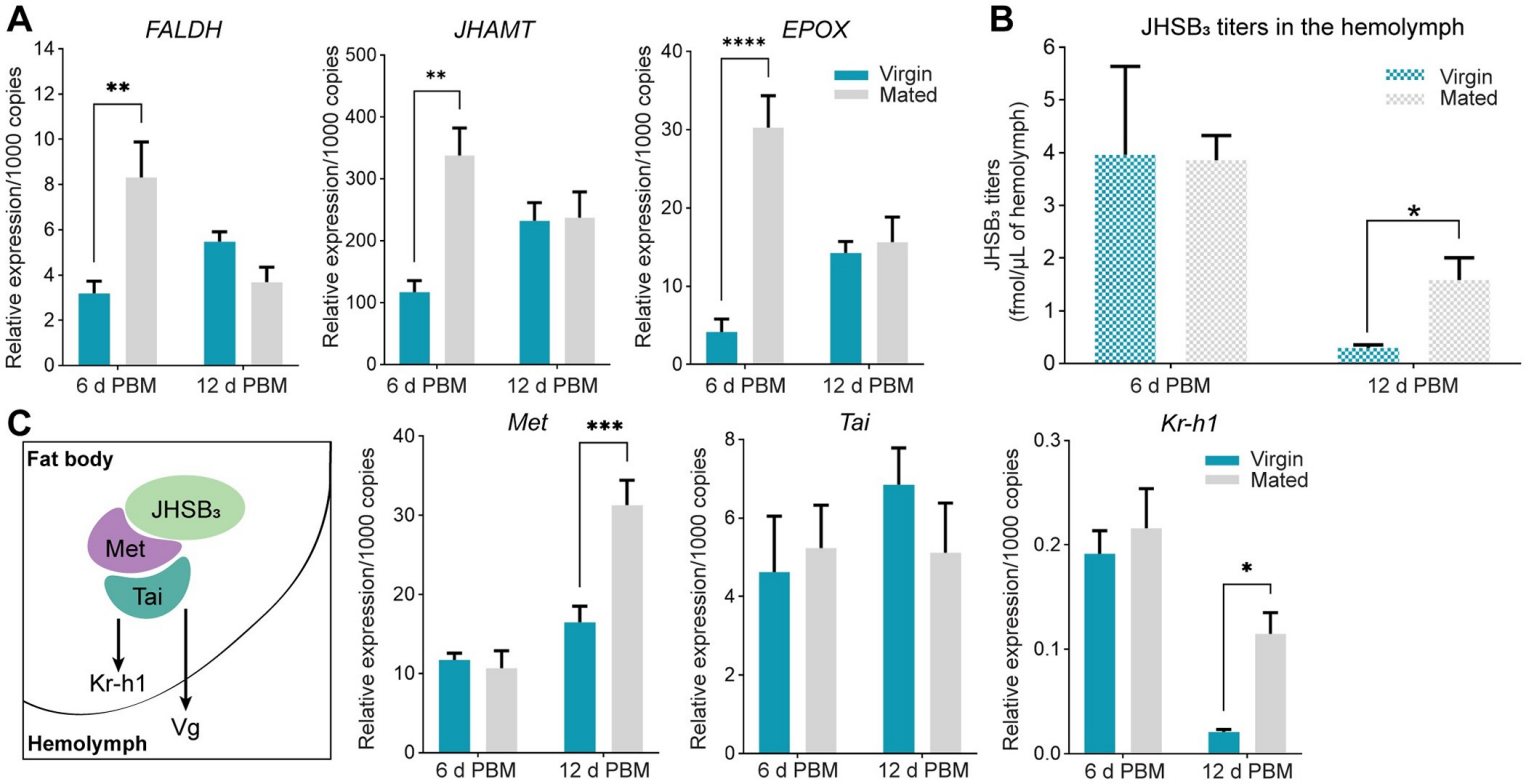
JHSB_3_ production in fed virgin and mated females of *R*. *prolixus*. **(A)** Transcript levels of three JH biosynthetic enzymes from the JH-branch were quantified in the CNS-CC-CA complexes from fed virgin and mated females at 6 and 12 days post blood meal (PBM) by RT-qPCR. The analysis was performed using the 2^−ΔCt^ method. The y axes represent the relative expression obtained via geometric averaging using *Rp49* and *18S* as reference genes. **p < 0.01; ****p < 0.0001 (Student’s t-test; n = 4 independent biological replicates, where each n represents a pool of CNS-CC-CA complexes from 3 insects). **(B)** Hemolymph was collected from fed virgin and mated females at 6 and 12 d PBM. The y axis represents JHSB_3_ titers (fmol) per μL of hemolymph. *p < 0.05; (Student’s t-test; n = 4–6 independent biological replicates, where each n is hemolymph from 5–7 insects). **(C)** Left: scheme of JHSB_3_-Met-Tai axis in the fat body modulating production of the downstream factor Kr-h1 and vitellogenin (Vg); right: transcript levels of *Met*, *Tai* and *Kr-h1* were quantified in the fat body from fed virgin and mated females at 6 and 12 days post blood meal (PBM) using RT-qPCR. The analysis was performed using the 2^−ΔCt^ method. The y axes represent the relative expression obtained via geometric averaging using *Rp49*, *18S* and *actin* as reference genes. *p < 0.05; ***p < 0.001 (Student’s t-test, n = 5–6 independent biological replicates, where each n represents an individual tissue from 1 insect). FALDH, farnesal dehydrogenase; JHAMT, juvenile hormone acid methyltransferase; EPOX, methyl farneseoate epoxidase.

The JH biosynthetic pathway includes 13 enzymatic reactions generally divided into early steps, which follow the mevalonate pathway (MVAP), and late steps, the JH-branch [33]. The juvenile hormone acid methyltransferase (JHAMT) enzyme has been proposed as the rate-limiting enzyme in JH biosynthesis, and its mRNA level and enzymatic activity are closely correlated with the patterns of JH synthesis [34,19,20]. In addition, the methyl farneseoate epoxidase (EPOX) enzyme seems to be important for improving JHs agonistic activity on the Met receptor [35]. Since the CA is the site of JH production, the impact of mating on mRNA expression of three enzymes of the JH-branch was studied in the central nervous system-*corpus cardiacum*-CA (CNS-CC-CA) complex. mRNA expression for farnesal dehydrogenase (*FALDH*), *JHAMT* and *EPOX*, are upregulated in mated females with respect to virgins at 6 days post blood meal (Fig 3A). To evaluate the mRNA expression of the biosynthetic enzymes with JH production, we measured the JHSB_3_ titers in the hemolymph of virgin and mated females. JHSB_3_ hemolymph titers at 6 days post blood meal are similar in both virgin and mated adult females (Fig 3B); however, at 12 days post blood meal, although the levels are lower compared to 6 days post blood meal, circulating JHSB_3_ is significantly higher in mated adult females relative to virgins (virgin vs. mated: 0.29 ± 0.09 fmol/μL vs. 1.58 ± 0.68 fmol/μL, Fig 3B). Recently, we reported that JHSB_3_ signaling, through Met and Tai, modulates transcript expression of downstream genes, such as *Kr-h1* and *Vg* [19] (Fig 3C). In the fat body, mRNA expression of *Met* and *Kr-h1* are upregulated in mated females at 12 days post blood meal with respect to virgins, while *Tai* transcript levels are not influenced by mating (Fig 3C).

### Mating influences transcript expression of ecdysteroid biosynthetic enzymes in the ovary, as well as hemolymph ecdysteroid titers

**Fig 4:**
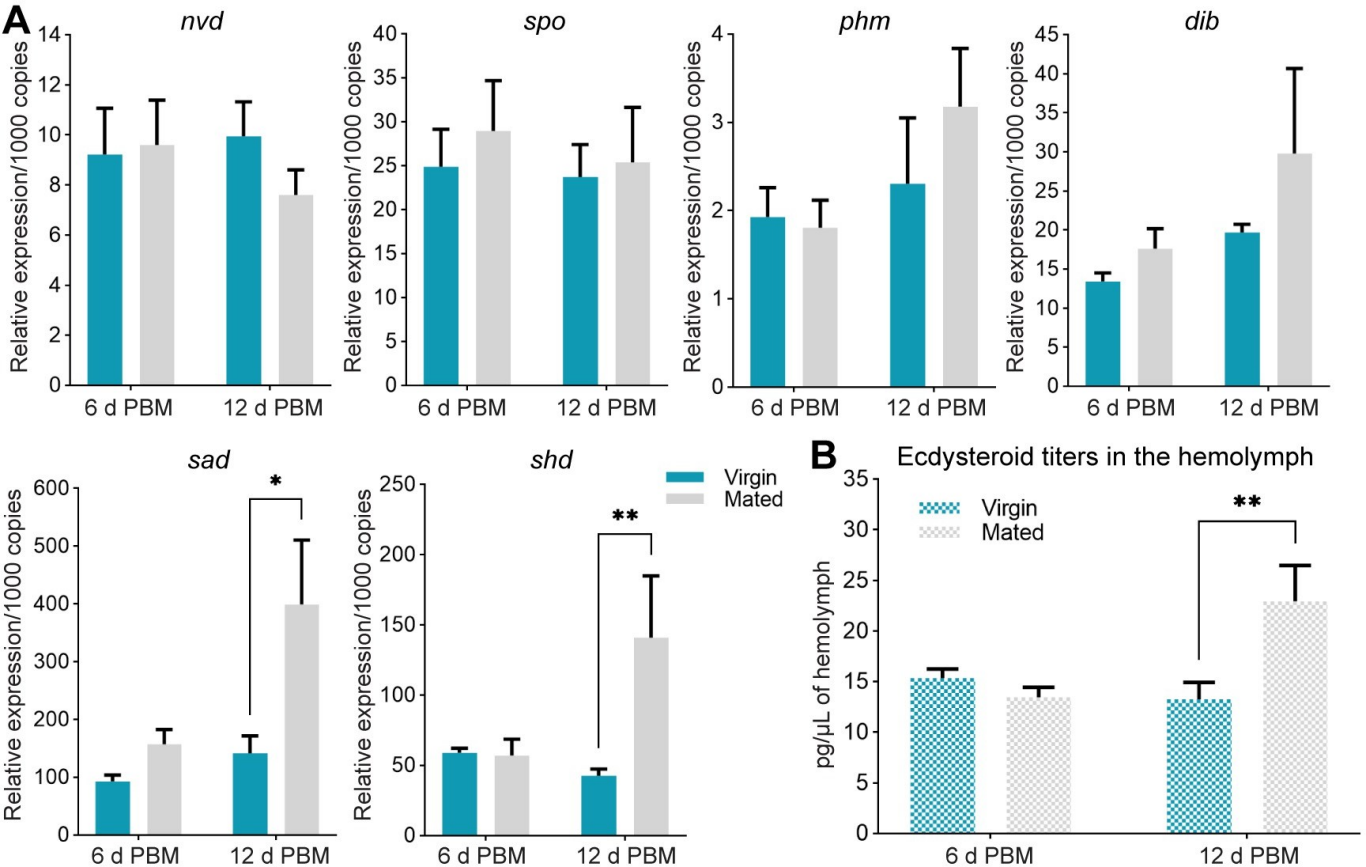
Ecdysteroid production in fed virgin and mated females of *R*. *prolixus*. **(A)** Transcript levels of ecdysteroid biosynthetic enzymes were quantified in the ovaries from fed virgin and mated females at 6 and 12 days post blood meal (PBM) by RT-qPCR. The analysis was performed using the 2^−ΔCt^ method. The y axes represent the relative expression obtained via geometric averaging using *Rp49* and *actin* as reference genes. *p < 0.05; **p < 0.01 (Student’s t-test; n = 4–5 independent biological replicates, where each n represents an individual tissue from 1 insect). **(B)** Ecdysteroid hemolymph titers of fed virgin and mated females at 6 and 12 PBM were quantified by ELISA. The y axis represents ecdysteroid titers (pg) per μL of hemolymph. **p < 0.001 (Student’s t-test, n = 4–5 independent biological replicates, where each n represents hemolymph from 1 insect). nvd, neverland; spo, spook; phm, phantom; dib, disembodied; sad, shadow; shd, shade.

The ecdysteroid biosynthetic pathway includes the rieske oxygenase neverland (nvd), and seven enzymes collectively known as the Halloween genes: non-molting glossy/shroud (sro), spook (spo), spookier (spok), phantom (phm), disembodied (dib), shadow (sad) and shade (shd), several of which have recently been identified in *R*. *prolixus* [23]. Here, we evaluated the potential effect of mating in regulating ecdysteroid biosynthesis in the ovaries of *R*. *prolixus* females. Mated females have an upregulation of transcripts expression of *sad* (*p* < 0.05) and *shd* (*p* < 0.01) at 12 days post blood meal (Fig 4A). When ecdysteroid titers are measured in the hemolymph at the same time point, a significant increase in mated females with respect to virgins is observed (virgin vs. mated: 13.25 pg/μL ± 0.98 vs. 22.95 ± 3.52 pg/μL, *p* < 0.01; Fig 4B).

### Mating decreases locomotory activity relative to virgins

**Fig 5:**
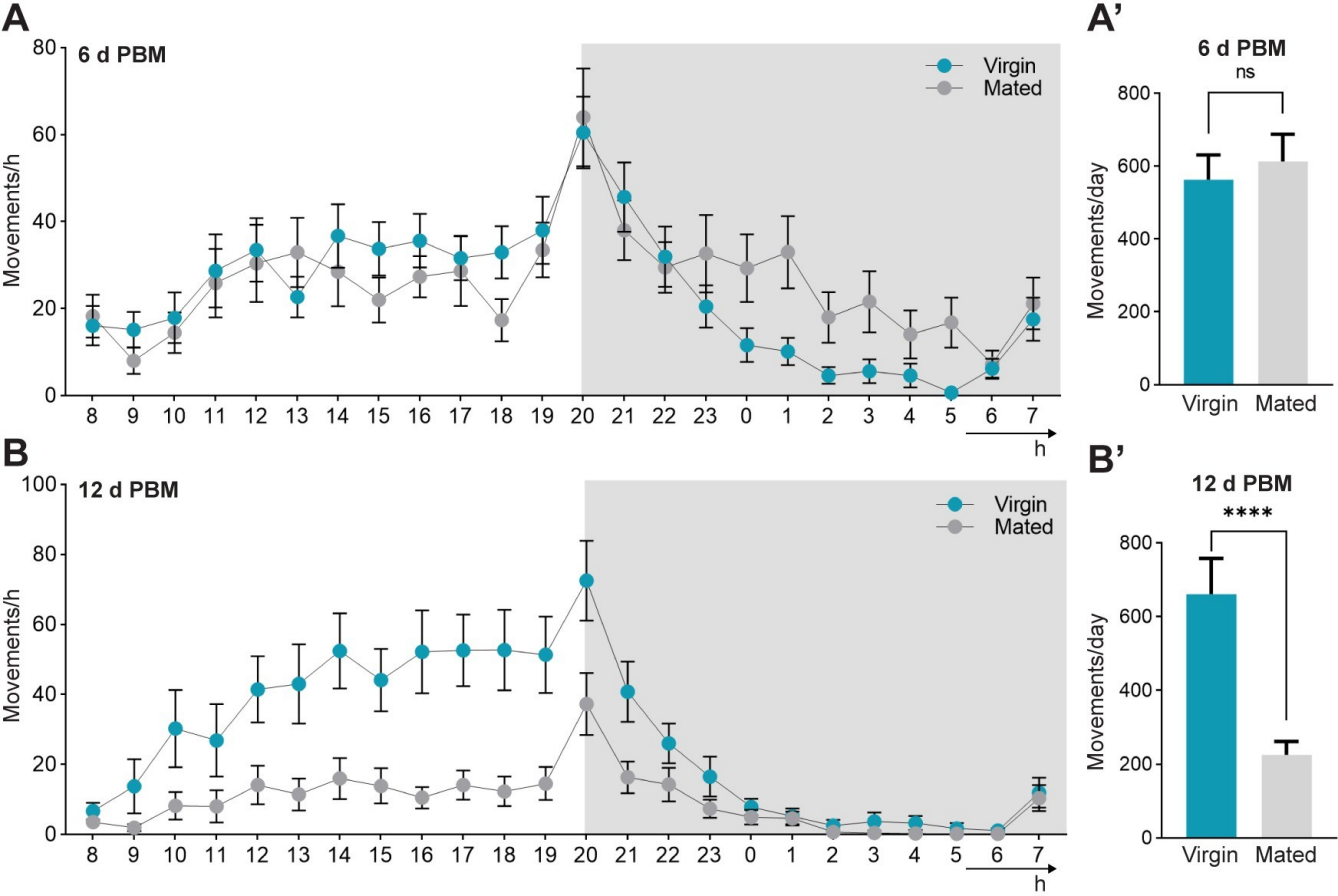
Locomotion of fed virgin and mated females of *R*. *prolixus*. **(A-B)** Fed virgin (blue symbols) and mated females (gray symbols) were individually placed inside actometer units and their movements recorded for 24 hours (*x* axis) at day 6 (A) and day 12 (B) post blood meal (PBM). Total number of movements of virgin and mated females 6 d PBM and 12 d PBM is shown in (A’) and (B’), respectively. White and grey areas depict the photophase and scotophase, respectively. Data are presented as the square root of the mean locomotory activity. ****p < 0.0001 (Student’s t-test, n = 35 insects for each treatment).

In addition to evaluating the effect of mating on metabolism and hormone profiles, we tested whether locomotory activity was also modified. The movements of virgin and mated insects were monitored over 24 h at days 6 and 12 post blood meal in a 12:12 h light/dark cycle. Actometer data show similar activity patterns at day 6 irrespective of female mating state, with one main peak at the beginning of the scotophase. Virgin females present a weaker activity during most of the scotophase, being significantly lower from that observed in mated females in hours 1, 3, 4 and 5 (Fig 5A and S1 Table; virgin vs. mated: 1 h, 3 h and 4 h, *p* = 0.02; 5 h, *p* = 0.002). Notwithstanding this slight modification of their activity profiles, no significant differences are observed in the total number of movements recorded at this post feeding time depending on female mating state (Fig 5A’, virgin vs. mated: 562.76 ± 68.08 movements/day vs. 613.1 ± 74.61 movements/day). Nevertheless, a significant decrease in the activity of mated females is observed during the photophase as well as in the beginning of the scotophase, on day 12 post blood meal (Fig 5B and S2 Table; virgin vs. mated: 12–21 h, *p* < 0.001; 22 h, *p* = 0.01). In this case, the total number of movements shown by virgin females is significantly higher (virgin vs. mated: 661.17 ± 97.16 movements/day vs. 225.8 ± 36.13 movements/day, *p* < 0.001; Fig 5B’).

## Discussion

Egg laying patterns of fed virgin and mated *R*. *prolixus* females were initially reported when a laboratory colony was established [36]. Several studies, including the pioneering work of Kenneth G. Davey, have examined the influence of mating on egg production [11,26,37–41]. However, it is still unclear how the signals received during mating are processed by females to modulate the biological responses that promote a full reproductive cycle. Here, we describe for the first time a comparative work showing the titers of two main insect hormones, JH and ecdysteroids, in virgin and mated females of *R*. *prolixus*. These titers appear to explain the differences seen in their reproductive performance. Finally, the decrease in activity seen in mated females at the peak of egg laying suggests an impact of energetic resource use on their spontaneous locomotory activity.

Recently, we reported very low JHSB_3_ levels in the hemolymph of unfed females [20]. Davey and Singleton [42] proposed that JH secretion between emergence of the adult and the first blood meal could prime the ovaries to respond immediately to the future feed. The blood meal represents the signal to promote the synthesis and release of higher amounts of JHSB_3_ into the circulation [20]. After feeding, two of the main functions of circulating JH are promoting vitellogenin synthesis in the fat body and opening intercellular spaces in the follicle epithelium (patency) [43]; processes that proceed differentially according to the relative JH titer [41]. In mated *R*. *prolixus* females, the JHSB_3_ titers after feeding are relatively constant until 6 days post blood meal, when egg laying begins [20]. Titers then decrease but are still 10-fold higher than those found in unfed females [20]. At 12 days post blood meal circulating JHSB_3_ titers decrease in both virgin and mated females but still remain significantly higher in mated females. This is supported in the virgins by a decrease in transcript levels of genes that have previously been reported to be regulated by JH signaling, such as *Vg* and *Kr-h1*, the latter being a proxy for JHSB_3_ titers in *R*. *prolixus* [19,20,34]. The metabolic activity, as judged by lipid and protein synthesis by the fat body, also diminishes in virgin females. Overall, our findings support the hypothesis that the CA ceases activity sooner in fed virgin females, explaining why egg production is reduced in these insects (see [11]). mRNA expression of JH biosynthetic enzymes in the CA has been correlated with the pattern of JHSB_3_ synthesis in *R*. *prolixus* females [20]. Here, we find that in mated insects JHSB_3_ titers are regulated by modulating transcript expression of the biosynthetic enzymes responsible for producing this hormone in the CA prior to the JHSB_3_ levels decreasing in the hemolymph.

Adult fed females of *R*. *prolixus* lay eggs in waves [26]; the first wave of egg production occurs in virgins as well as mated females, but in the former group, it is completed at a slower pace, with the eggs accumulating in the ovaries. In adults, ecdysteroids are synthesized by the ovaries and then released into the hemolymph [23,44,45]. One of the early actions of hemolymph ecdysteroids in mated *R*. *prolixus* females is their involvement, along with a mating factor, in the release of a myotropic neurohormone from MNSCs in the brain. This myotropin then stimulates ovulation and possibly oviposition [11]. Here, we show that between days 6 and 12 post blood meal, circulating ecdysteroid titers only increase in mated females, being stable in virgins. Thus, this hormonal network, which includes a lower ecdysteroid titer, the absence of the mating factor and the myotropin, is likely responsible for the delayed egg laying seen in virgins. The absence of the mating factor results in myotropin not being released, and therefore ovulation/oviposition are delayed. Virgin *R*. *prolixus* females fail to successfully carry out the second wave of egg laying [11,15]. As is known, at the end of the first reproductive cycle, virgins have made and laid less eggs than mated females, with the difference in production being eliminated by the application of exogenous JH [46]; suggesting that the decrease in reproductive output is mainly due to lower JH titers in virgins. Our studies confirm that JHSB_3_ titers are lower in virgin females at 12 days post blood meal when compared to mated females.

The fat body in *R*. *prolixus* is primarily an organ of intermediary metabolism, and the AMG (often refereed to as the crop) has been defined as the main organ of blood meal storage. The ingested blood is composed mainly of proteins, and other nutrients, such as carbohydrates and lipids, in relatively low amounts [47]. The rate of digestion of the intestinal contents has been positively correlated with egg production [48]. In fed virgin females of *R*. *prolixus*, the absence of a mating factor might be a signal to the CA to stop JH synthesis, and thus avoid the production of infertile eggs. In addition, the blood stored in the AMG is released into the PMG more slowly by virgin females, thereby retaining nutrients for future use. This fact can be considered a biological strategy allowing virgin females to survive longer in order to increase the chances of successful reproduction. In support of this hypothesis, we show that the survival rate at 100 days after a blood meal intake is at least 3-fold higher in virgin females. However, we should keep in mind that the shorter lifespan of mated females may be a consequence of the excessive use of energy to make eggs (a reproductive cost); indeed, this is the most prevalent hypothesis on the relationship between reproduction and longevity [28]. Trade-offs may arise because of a direct physiological conflict between reproduction and longevity since both processes are related to nutrition. In addition, several pathways are emerging as being important in defining reproductive cost, such as insulin signaling, nutrient sensing, and immunity [49]. Also, the low survival rate of mated *R*. *prolixus* females can be a consequence of male sexual harassment, potential multiple mating, toxic side effects of male ejaculate, and/or a hormone environment inducing vulnerability in the immune system [28,50,51].

Locomotion is an active process by which insects seek resources, such as food and mates, and refuge from enemies [52]. Triatomines are more active at night, showing a strong negative phototaxis that directs them to shelters, a behaviour that allows them to increase their chances of survival against predators [53]. Interestingly, we found that *R*. *prolixus* females also move during daylight when recently fed, suggesting that their spontaneous locomotion, i.e., activity in absence of host cues, can be related to positive energetic reserves. This idea is reinforced by the significantly reduced activity of mated females in relation to virgins at day 12 post blood meal. The much larger egg production shown by mated females requires intensive use of nutrients, possibly resulting in their limited locomotion once egg production is at its peak. It is worth mentioning that locomotion induces a significant loss of mass in females through an increase in both metabolic rate and water loss [54]. The decreased spontaneous activity seen in mated females may represent a resource saving strategy, since a significant part of the blood meal has already been committed to egg production. The hypothesis that locomotory activity is related to nutrient reserves has also recently been suggested in triatomines [55]. Overall, the low level of activity seen for females that have produced more than half of their first egg batch, i.e. mated females fed 12 days earlier, may represent a novel reproductive trade-off [28,50,51]. As such, we suggest that our work also depicts significant costs of reproduction faced by triatomine females.

Overall, this work highlights molecular, biochemical, hormonal and behavioral features, possibly initiated by the mating factor, which rapidly switches females from a virgin to a mated state. This research opens doors for future studies to define specific factors that initiate each of these physiological responses. This study is critically important not only to improve our understanding of insect physiology but also to provide options for translational research that could generate novel strategies for pest management.

## Materials and methods

### Experimental animals

*R*. *prolixus* were taken from long-established colonies at the University of Toronto Mississauga and from the Vector Behaviour and Pathogen Interaction Group at the René Rachou Institute [56]. Insects were reared and kept in incubators in wide mouth pint glass jars (capacity 500 ml), at 25°C and 50 ± 5% humidity and artificially fed on sterile defibrinated rabbit blood as detailed by Orchard et al. [29]. The glass jars contain a single flat piece of filter paper on the bottom and pleated filter paper acting as vertical surfaces, thereby mimicking the crevices that insects normally inhabit and on which insects can rest and oviposit. For the experiments, insects were sorted by sex during the last nymphal instar, by examination of the final abdominal tergites and sternites under a dissecting stereo microscope. Thirty days post ecdysis, insects were fed *ad libitum*. To prepare the experimental group of mated insects, newly emerged adult females were segregated individually and placed together with two recently fed males. Mating was verified by examining the cubicle for spermatophores. In order to promote the initiation of the first reproductive cycle, 10 day-old virgin and mated females were fed, and only those that gorged 2.5 to 3 times their initial body weight were used for experiments. They were later placed in individual transparent cubicles (2 cm x 3 cm) and maintained in a 12/12 h light/dark cycle. Laid eggs were recorded every 2 days through a 28 day interval post blood meal. Female mortality rates were recorded during 100 days post blood meal. A group of virgin and mated animals was dissected in cold autoclaved phosphate-buffered saline (PBS, 8.2 mM Na_2_HPO_4_, 1.5 mM KH_2_PO_4_, 150 mM NaCl, 2.7 mM KCl) at day 6 and 12 post blood meal and female reproductive systems were photographed using a Leica DVM6 digital microscope (Leica Microsystems, Wetzlar, Germany). In order to avoid differences due to circadian rhythm, all dissections were performed during the first 2 hours of the scotophase.

### Protein analysis: SDS-PAGE, Western blot and ELISA

Virgin and mated females were dissected in cold autoclaved PBS. The AMG of each insect (including luminal contents) was carefully collected by cutting off at the end of the foregut and at the juncture with the posterior midgut, blotted and immediately placed into a pre-weighed microtube containing 200 μL of cold lysis buffer; thereafter, microtubes were re-weighed in a microbalance. For cell lysis and total protein extraction, the fat body and AMG were individually homogenized in 200 μL of cold, freshly-made lysis buffer RIPA buffer [150 mM NaCl, 1% Triton X-100, 0.5% sodium deoxycholate, 0.1% SDS, 50 mM Tris, pH 8.0 in double-distilled or MilliQ water] plus 2 μL of protease and phosphatase inhibitor cocktails (P5726 and P8340, respectively, Sigma-Aldrich, ON, Canada). The homogenates were then centrifuged at 17,000 × g for 25 min at 4°C. Ten μL of hemolymph was collected from virgin and mated females with a manual pipette 0.1–10 μL by cutting off the legs at the level of the coxa, and gently pressing the abdomen. Hemolymph was placed in ice-cold tubes containing 2 μL of anticoagulant solution (10 mM EDTA, 26 mM sodium citrate, 26 mM citric acid and 100 mM glucose in PBS). Hemolymph samples were then centrifuged at 15,000 × g for 10 min at 4°C to remove hemocytes. Protein quantifications of all the resulting infranatants were performed using the BCA protein quantification assay (Pierce BCA Protein Assay Kit by Thermo Fischer, ON, Canada).

Protein bands from 1 μL hemolymph of virgin and mated females were separated under reducing conditions on pre-made gels (percentage 4–20%, Mini-Protean TGX Stain-Free Precast Gels, BioRad, Mississauga, ON, Canada). The gels were then stained with QC Colloidal Coomassie (BioRad), for 1 h at room temperature. After washing, gels were imaged on a ChemiDoc XRS system (BioRad). For Western blot, 1 μL of hemolymph (1:20 dilution) was used to separate protein under reducing conditions on pre-made gels. A detailed protocol for Western blot was previously described [19]. Briefly, the polyclonal anti-Vg antibody was commercially acquired from Boster Biological Technology (Pleasanton, CA, USA). Primary antibody (dilution 1:2000) was incubated overnight at 4°C. A horseradish peroxidase (HRP) conjugated goat anti-rabbit IgG (secondary antibody, dilution 1:5000) was then incubated for 1 h at room temperature. Blots were visualized using enhanced chemiluminescence (Clarity Western ECL Substrate, BioRad). The images shown are representatives of 3 independent experiments.

Quantification of Vg in the hemolymph was carried out by the enzyme-linked immunosorbent assay (ELISA) as described by Leyria et al. [19]. Briefly, microtiter plates were loaded with 200 μL/well of recombinant Vg (Boster Biological Technology) or with appropriate hemolymph dilutions in buffer carbonate (15 mM Na_2_CO_3_, 35 mM NaHCO_3_, pH 9.6), incubated for 90 min at 37°C and then washed with PBST (0.05% Tween 20 in PBS). Next, wells were incubated with anti-Vg antibody (0.01 μg/mL, in 0.1% of bovine serum albumin-PBST), with anti-rabbit immunoglobulin conjugated to HPR in PBST (1:5000; 30 min at 37°C) and finally with TMB Liquid Substrate System (Millipore-Sigma, Oakville, ON, Canada). Plates were read at 492 nm using a multi-mode reader (Synergy HTX from Agilent Technologies, Santa Clara, CA, USA). A standard curve using the recombinant antigen sequence (from 50 pg/mL to 10 mg/mL) was performed in parallel. The results are shown as the mean ± SEM of n = 5–6 independent biological replicates, where each n represents hemolymph from 1 insect.

### Lipid quantification

For lipid determination, ventral and dorsal fat bodies from virgin and mated females 6 and 12 days post blood meal were dissected under cold PBS. Tissues were placed in 500 μL of isopropanol, homogenized and then centrifuged at 8,000 x g for 10 min at 20°C. Four-hundred μL of the supernatants were transferred to tubes containing 100 μL of 1 M KOH. In addition, 5 μL of hemolymph was collected from each insect and immediately placed in 50 μL of 10% trichloroacetic acid and centrifuged at 8,000 × g for 5 min at 20°C. Pellets containing lipids associated with lipoproteins were resuspended in 400 μL isopropanol plus 100 μL of 1 M KOH. Lipid level measurements in the fat body and hemolymph were analysed using a colorimetric assay, as previously detailed [57]. Briefly, the tubes containing all the samples were incubated at 60°C for 10 min and then 100 μL of sodium periodate solution (11.6 mM sodium periodate in 2 N glacial acetic acid) was added. Afterward, 600 μL of chromogenic solution (40 mL of 2 M ammonium acetate, 40 mL of isopropanol, 150 mL of acetyl acetone) were aggregated and the resultant colour was measured at 410 nm using a plate reader spectrophotometer (Cytation 3 Imaging Reader, BioTek, Winooski, VT, USA). A curve of commercial triglycerides ranging from 0 to 60 μg was run in parallel with the experimental samples. The results are shown as the mean ± SEM of n = 5–6 independent biological replicates, where each n represents hemolymph or fat body from 1 insect.

### RNA extraction and reverse transcription/quantitative PCR (RT-qPCR)

Ventral and dorsal fat bodies, ovaries and the CNS-CC-CA from virgin and mated females 6 and 12 days post blood meal were dissected under cold autoclaved PBS. Total RNA was extracted from tissues using TRIzol reagent (Invitrogen by Thermo Fisher Scientific, Waltham, MA, USA) according to the manufacturer’s instructions, and then subjected to DNase treatment using DNase I (RNase-free) Kit (Thermo Fisher Scientific, Mississauga, ON, Canada), as detailed by [20]. Synthesis of first-strand complementary DNA (cDNA) was done using the Applied Biosystems High Capacity cDNA Reverse Transcription Kit (Applied-Biosystems, by Thermo Fisher Scientific, Mississauga, ON, Canada). Quantitative PCR was performed using an advanced qPCR 1-Step Kit with Supergreen Dye Low-ROX (Wisent Bioproducts Inc., Saint-Jean-Baptiste, QC, Canada) using *β-actin*, *ribosomal protein 49* (Rp49) or *18S ribosomal RNA* (18S) as reference genes (primers are shown in S3 Table). mRNA expression was calculated relative to 1000 copies of the average of the reference genes using the 2^−ΔCt^ method [58]. Experiments were repeated with at least five independent biological replicates, as indicated in each experiment, having two technical replicates and using no-template controls.

### JHSB_3_ and ecdysteroid quantification in hemolymph

The JHSB_3_ levels present in the hemolymph from virgin and mated females at 6 and 12 days post blood meal were quantified employing a liquid chromatography coupled to tandem mass spectrometry protocol, as previously described [59]. The JHSB3 amounts present in the hemolymph were quantified using a deuterated JH III analog (JH III-D3, from Toronto Research Chemicals, North York, ON, Canada) as an internal standard [60]. Briefly, 70 μL of hemolymph were collected for each female and placed in cold glass silanized vials (Thermofisher Scientific, Waltham, MA, USA) containing 60 μL of anticoagulant solution. After that, 10 μL of 6.25 ppb of JH III-D3 in acetonitrile were added to each sample, followed by 600 μL of hexane. Samples were mixed and then centrifugated. The organic phase was transferred to a new silanized vial and dried under nitrogen flow. Dried extracts were resuspended in 50 μL of acetonitrile and transferred to a vial with a fused 250 μL insert. JHSB_3_ quantification was based on multiple reaction monitoring (MRM), using the two most abundant fragmentation transitions: 283 → 233 (primary) and 283 → 145 (secondary) [59].

The ecdysteroid levels present in the hemolymph, taken from the same females as above, were quantified by competitive ELISA as described by Leyria et al. [20]. Briefly, 5 μL of hemolymph were combined with methanol at a ratio (1:3) (sample:methanol), centrifuged, and vacuum dried. The samples were then resuspended using assay buffer (25 mmol l^−1^ sodium phosphate, pH 7.5; 150 mmol l^−1^ NaCl; and 1 mmol l^−1^ EDTA disodium dehydrate) containing 0.1% bovine serum albumin (BSA, faction V, Heat shock isolation, Bioshop, ON, Canada) and the competitive ELISA carried out using 20E conjugated to HRP reagent and a rabbit anti-ecdysteroid primary antibody as detailed by Abuhagr et al. [61].

### Locomotory activity analysis

We used an automatic actometer system consisting of 40 individual arenas (10 x 5 x 2 cm), each presenting three equally distant light barriers (a light emitting diode (LED) and a phototransistor), to record the activity of virgin and mated females. Thirty-five virgin and mated females (same number for each group) were placed individually in the actometer. After an initial 24 h acclimation interval, female activity was recorded during 24 h (12/12 h light/dark cycle). The same procedure was applied to all females at 6 and 12 days after feeding. Each time an insect movement interrupted one of the light beams, the system generated a signal recorded by an ad hoc software [62]. Each arena had a filter paper as substrate and was covered with a rectangular acrylic lid. The device was set up inside a controlled environment chamber (26 ± 2°C; 50 ± 5% RH, light intensity = 60 lux).

### Statistical analysis

All data were processed using the GraphPad Prism 9 Software (GraphPad Software, San Diego, CA, USA). Results are shown as mean ± SEM. All datasets passed normality and homoscedasticity tests. Significance of differences were determined either with one-tailed Student’s t-test, or with one-way ANOVA followed by Tukey’s test, as indicated. When parameters studied at 6 and 12 days post blood meal are shown in the same graph, only columns of the same day were compared using Student’s t-test. The Wilcoxon signed-rank test (paired samples T-Test) was used to compare the activity of every single hour between virgin and mated females.

## Supporting information

S1 Table Result of Wilcoxon signed-rank test for locomotion of fed females during 24 h at 6 d PBM. *p* > 0.05 are highlighted. m, mated females; v, virgin females.
(DOCX)

S2 Table Result of Wilcoxon signed-rank test for locomotion of fed females during 24 h at 12 d PBM. *p* > 0.05 are highlighted. m, mated females; v, virgin females.
(DOCX)

S3 Table Primers used for qPCR (previously reported by [19,20]). (DOCX)
