# Supplementary material for: Effects of mating on female reproductive physiology in the insect model, Rhodnius prolixus, a vector of the causative parasite of Chagas disease
Source: PLoS Negl Trop Dis. 2023 Sep 20;17(9):e0011640. doi: 10.1371/journal.pntd.0011640 (PMC10545099; doi:10.1371/journal.pntd.0011640)
Supplement: S1 Table — p > 0.05 are highlighted. m, mated females; v, virgin females. (DOCX) [file pntd.0011640.s001.docx]

**S1 Table.** Result of Wilcoxon signed-rank test for locomotion of fed females during 24 h at 6 d PBM. *p* > 0.05 are highlighted. m, mated females; v, virgin females.

| **Measure (m)** | | **Measure (v)** | | **W** | | ***p*** | |
| --- | --- | --- | --- | --- | --- | --- | --- |
| 07m6 |  | 07v6 |  | 250.000 |  | 0.489 |  |
| 08m6 |  | 08v6 |  | 212.000 |  | 0.846 |  |
| 09m6 |  | 09v6 |  | 133.000 |  | 0.286 |  |
| 10m6 |  | 10v6 |  | 111.500 |  | 0.638 |  |
| 11m6 |  | 11v6 |  | 169.500 |  | 0.889 |  |
| 12m6 |  | 12v6 |  | 178.500 |  | 0.585 |  |
| 13m6 |  | 13v6 |  | 322.000 |  | 0.464 |  |
| 14m6 |  | 14v6 |  | 217.500 |  | 0.557 |  |
| 15m6 |  | 15v6 |  | 157.000 |  | 0.194 |  |
| 16m6 |  | 16v6 |  | 216.000 |  | 0.537 |  |
| 17m6 |  | 17v6 |  | 212.000 |  | 0.487 |  |
| 18m6 |  | 18v6 |  | 154.000 |  | 0.067 |  |
| 19m6 |  | 19v6 |  | 294.500 |  | 0.966 |  |
| 20m6 |  | 20v6 |  | 322.500 |  | 0.675 |  |
| 21m6 |  | 21v6 |  | 271.500 |  | 0.663 |  |
| 22m6 |  | 22v6 |  | 255.000 |  | 0.899 |  |
| 23m6 |  | 23v6 |  | 274.000 |  | 0.399 |  |
| 00m6 |  | 00v6 |  | 307.000 |  | 0.054 |  |
| 01m6 |  | 01v6 |  | 266.000 |  | 0.022 |  |
| 02m6 |  | 02v6 |  | 211.000 |  | 0.084 |  |
| 03m6 |  | 03v6 |  | 211.000 |  | 0.027 |  |
| 04m6 |  | 04v6 |  | 138.000 |  | 0.023 |  |
| 05m6 |  | 05v6 |  | 242.000 |  | 0.002 |  |
| 06m6 |  | 06v6 |  | 195.000 |  | 0.629 |  |
