# Supplementary material for: Effects of mating on female reproductive physiology in the insect model, Rhodnius prolixus, a vector of the causative parasite of Chagas disease
Source: PLoS Negl Trop Dis. 2023 Sep 20;17(9):e0011640. doi: 10.1371/journal.pntd.0011640 (PMC10545099; doi:10.1371/journal.pntd.0011640)
Supplement: S2 Table — p > 0.05 are highlighted. m, mated females; v, virgin females. (DOCX) [file pntd.0011640.s002.docx]

**S2 Table.** Result of Wilcoxon signed-rank test for locomotion of fed females during 24 h at 12 d PBM. *p* > 0.05 are highlighted. m, mated females; v, virgin females.

| **Measure (m)** | | **Measure (v)** | | **W** | | ***p*** | |
| --- | --- | --- | --- | --- | --- | --- | --- |
| 07m |  | 07v |  | 216.500 |  | 0.516 |  |
| 08m |  | 08v |  | 72.500 |  | 0.376 |  |
| 09m |  | 09v |  | 43.000 |  | 0.348 |  |
| 10m |  | 10v |  | 86.000 |  | 0.069 |  |
| 11m |  | 11v |  | 78.500 |  | 0.072 |  |
| 12m |  | 12v |  | 60.500 |  | 0.001 |  |
| 13m |  | 13v |  | 71.000 |  | 0.008 |  |
| 14m |  | 14v |  | 70.500 |  | 8.936e-4 |  |
| 15m |  | 15v |  | 99.000 |  | 0.004 |  |
| 16m |  | 16v |  | 66.000 |  | 3.745e-4 |  |
| 17m |  | 17v |  | 46.000 |  | 7.848e-5 |  |
| 18m |  | 18v |  | 67.500 |  | 2.470e-4 |  |
| 19m |  | 19v |  | 94.000 |  | 0.003 |  |
| 20m |  | 20v |  | 115.500 |  | 0.002 |  |
| 21m |  | 21v |  | 84.500 |  | 0.001 |  |
| 22m |  | 22v |  | 103.000 |  | 0.014 |  |
| 23m |  | 23v |  | 117.000 |  | 0.533 |  |
| 00m |  | 00v |  | 60.500 |  | 0.286 |  |
| 01m |  | 01v |  | 68.500 |  | 0.722 |  |
| 02m |  | 02v |  | 25.000 |  | 0.503 |  |
| 03m |  | 03v |  | 36.000 |  | 0.308 |  |
| 04m |  | 04v |  | 28.000 |  | 0.681 |  |
| 05m |  | 05v |  | 20.000 |  | 0.809 |  |
| 06m |  | 06v |  | 27.500 |  | 0.118 |  |
