# Supplementary material for: Effects of mating on female reproductive physiology in the insect model, Rhodnius prolixus, a vector of the causative parasite of Chagas disease
Source: PLoS Negl Trop Dis. 2023 Sep 20;17(9):e0011640. doi: 10.1371/journal.pntd.0011640 (PMC10545099; doi:10.1371/journal.pntd.0011640)
Supplement: S3 Table — (DOCX) [file pntd.0011640.s003.docx]

**S3 Table.** Primers used for qPCR (previously reported by [1] (^§^) and [2] (*))

| **Gene code** | **Primers to qPCR** | **Sequence (5🡪3)** |
| --- | --- | --- |
| JN416985.1 | Total Met_forward | GATGGGAGGAATCCTGTTGA |
|  | Total Met_reverse | GGCAAGTCTGGATCGAAGTC |
| RPRC001318; RPRC001317 | Tai_forward | CACAACGTCCATCCACTCCA |
|  | Tai_reverse | TTCTTTGCAGCGGTCTCACT |
| RPRC014398 | Kr-h1_forward | ACAACCTGTAGTGGCTGTCG |
|  | Kr-h1_reverse | CGTACACTGTAGCGTGTCGT |
| RPRC002109 | Vg 2_forward | TCCATTGCCTAACCTCCTTG |
|  | Vg 2_reverse | GTAAGGACGATGCGGCTAAC |
| RPRC013511 | Vg 1_forward | TTGCTAGTCGCATGAACCTG |
|  | Vg 1 _reverse | TTTAGTGGTGCATCGCTCTG |
| RPRC009875 | Actin_forward | AGAGAAAAGATGACGCAGATAATGT |
|  | Actin _reverse | ATATCCCTAACAATTTCACGTTCG |
| RPRC014419 | Rp49_forward | GTGAAACTCAGGAGAAATTGGC |
|  | Rp49_reverse | AGGACACACCATGCGCTATC |
| RPRC011659 | ^§^JHAMT_forward | GGACCAGGCGATGTTACTTT |
|  | JHAMT_reverse | CCAAATCATCAGAAATATCGCTTCC |
| RPRC000513 | ^§^EPOX_forward | CGGAGAATTGATTCATGATGATTGG |
|  | EPOX_reverse | GTAACGGCGGTGACAGTAAA |
| RPRC002910 | ^§^FALDH_forward | AGTACCTTACAGTCTAGTATTTGCC |
|  | FALDH_reverse | GATCTGTCTTCAGCACCGTT |
| RPRC011241 | *Spook_forward | TGGCATTCTCCGATTGGTCT |
|  | Spook_reverse | TCATTGAGCAACGTGTCCAGT |
| RPRC006417 | *Shadow_forward | GAGGCAAGTTTTCGAAGTGG |
|  | Shadow_reverse | TGATTCATAATTCGGCGATG |
| RPRC011595 | *Dib_forward | TTGCACACTACCGTTTGTCG |
|  | Dib_reverse | AATGCGAGCAAGTGGTTTTT |
| RPRC009372 | *Phantom_forward | TGCCATACACGGAAGCATGT |
|  | Phantom_reverse | CGATAGCCTGCCAGTTCAGT |
| RPRC006945 | *Shade_forward | ATTCTTTGGGCTCCCATTCT |
|  | Shade_reverse | GCCATGAACACACTTTGCAC |
| RPRC001631 | *Nvd_forward | TGCTCCACTCATCTTGAAAGC |
|  | Nvd_reverse | ACTTGGCAATGGTTTTGTCTGT |

**References**

1. Leyria J, Orchard I, Lange AB. Impact of JH signaling on reproductive physiology of the classical insect model, *Rhodnius prolixus*. Int J Mol Sci. 2022;23: 13832.
2. Leyria J, Benrabaa S, Nouzova M, Noriega FG, Tose V, Fernandez-Lima F, et al. Crosstalk between nutrition, insulin, juvenile hormone, and ecdysteroid signaling in the classical insect model*, Rhodnius prolixus*. Int J Mol Sci. 2023;24: 7.
